# Emergency medicine in Saudi Arabia: a century of progress and a bright vision for the future

**DOI:** 10.1186/s12245-019-0232-0

**Published:** 2019-07-08

**Authors:** Eyad Khattab, Abdulrahman Sabbagh, Nawfal Aljerian, Hashim Binsalleeh, Mobarak Almulhim, Abdulrahman Alqahtani, Majid Alsalamah

**Affiliations:** 1 Harvard Medical School, Department of Emergency Medicine, Beth Israel Deaconess Medical Center, One Deaconess Road, WCC2, Boston, MA 02215 USA; 2 Department of Emergency Medicine, College of Medicine, King Saud University, Riyadh, Saudi Arabia; 3 Department of Emergency Medical Services, College of Applied Medical Sciences, King Saud Bin Abdulaziz University for Health Sciences, Ministry of Health, Riyadh, Saudi Arabia; 4 Prince Sultan bin Abdulaziz College for Emergency Medical Services, King Saud University, Riyadh, Saudi Arabia; 5 Department of Emergency Medicine, King Fahad Medical City, Riyadh, Saudi Arabia; 6 Department of Emergency Medicine, King Saud Medical City, Riyadh, Saudi Arabia; 7 Royal Clinics of the Custodian of the Two Holy Mosques, Riyadh, Saudi Arabia

**Keywords:** Emergency medicine, Saudi Arabia, International health, Global medicine, Global Health

## Abstract

Although emergency medical services (EMS) and pre-hospital care have existed in the Kingdom of Saudi Arabia (KSA) since 1934, emergency medicine (EM) is a relatively new medical field in the country that was not formally recognized as a medical specialty until 2001. In 2005, the Saudi Board of Emergency Medicine formed to develop, implement, and evaluate a standardized curriculum for EM residents. Since then, EM and the pre-hospital system in the KSA has evolved and grown. This article provides an overview of emergency medicine in Saudi Arabia and the progress it has made in the pre-hospital system, healthcare delivery system, and emergency medicine training. Finally, we will discuss the challenges and opportunities faced as this specialty continues to develop.

## Introduction

The Kingdom of Saudi Arabia (KSA) is the fifth largest country in Asia and the second in the Arab world in terms of area [1, 2] and has a population estimated at 32.5 million as of 2016 [3]. Due to the dry and arid desert terrain, 65% of the population live in just 3 of the 13 administrative regions: Makkah, Riyadh, and the Eastern Province. Despite its wealth, large size, centralized government, and sizable population clustered around urban areas, the KSA has a relatively new emergency medicine (EM) system. To better understand the current state of EM, as well as where it came from and where its headed, this article provides an overview of the development of EM in the KSA in addition to the challenges and opportunities faced as this important specialty continues to develop.

**Fig. 1:**
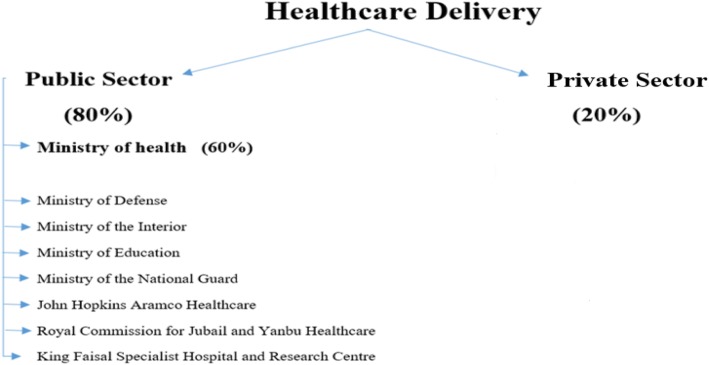
Breakdown of the healthcare system in Saudi Arabia

Healthcare in the KSA is delivered primarily through a publicly funded health system accounting for 80% of the healthcare provided in the country through more than 3000 primary care centers spread across the KSA. The Ministry of Health (MoH) is the main provider, operating around 60% of the hospitals and primary care centers. Other governmental agencies, including the Ministry of Defense (MoD), the Ministry of National Guard (MNG), the Royal Commission for Jubail and Yanbu, the Ministry of Education, and the Ministry of the Interior (MoI) provide the other 20% of publicly funded healthcare, mostly to predefined beneficiaries such as their employees and their dependents [4]. The remaining 20% of healthcare delivery in the KSA is provided by the private sector. Emergency access is guaranteed for everyone, including expats and visitors (see Fig. 1).

The government funds the public healthcare system via the parenting ministry (e.g., MoH, MoD, or MoE) to the corresponding health system. In addition to its role in financing and delivering healthcare, the MoH, with a budget of nearly 6 billion SR ($1.6 billion USD), is responsible for the country’s public health, policy and planning, and regulation. In line with Saudi Vision 2030, a roadmap for social and economic development in the KSA, the MoH is working with other governmental agencies to change the way healthcare is delivered.

## Pre-hospital emergency medical service (EMS)

### A brief history

Healthcare in Saudi Arabia predates the establishment of the modern state known as the KSA, with most of the early development occurring in Makkah because of the frequent medical needs experienced by the Hajj and Umrah pilgrims flocking to the holy city from around the world. A public health department, based in Makkah, was established in 1925. The first school of nursing was opened in 1926, followed by the School of Health and Emergences in 1927 [5]. In 1932, the KSA was internationally recognized as a nation and, 2 years later, the first ambulance service under the name of Ambulance Charity Association was created to serve pilgrims to the holy sites of Makkah and Madinah. It included a station and few ambulances [4, 5] to transport exhausted, injured, and sick pilgrims to the Ajyad Hospital, the only hospital at that time in Makkah [6]. It was primarily funded by charitable donations from locals and visitors, with some from the government as well. It was operated by volunteers and managed by a council who planned, budgeted, and organized the service. In 1951, the KSA established the MoH and, in 1963, the Saudi Red Crescent Association (SRCA) was established to provide the pre-hospital care in the country (changing its name to the Saudi Red Crescent Authority in 2008) [6]. With better funding and organization, pre-hospital care progressed in its infrastructures, personnel, and coverage.

While some developments occurred from 1934 to 2005, EMS education for first responders and emergency medical technicians (EMTs) during this period was limited to basic courses and workplace training [7]. In 2005, EMS diploma programs for basic and intermediate EMTs began in both public and private institutes. EMS in the KSA grew as a profession in this period, particularly when the Saudi Commission for Health Specialties (SCFHS) officially recognized and registered it. In 2012, the KSA mandated a minimum of a bachelor degree to work in any healthcare field Armed Forces Medical Services [8]. Since then, EMS programs have increased in number and quality.

### EMS Service

Pre-hospital emergency response is provided by a third-party service model fully operated by the SRCA, the 91st member of the International Federation of Red Cross and Red Societies [7]. The SRCA has a dedicated call number (997), unlike other public safety agencies in the KSA, and is fully funded by the government, with a 2016 annual budget of 568 million USD [9]. The SRCA has 404 stations across the KSA, an estimated 2000 ambulances and service vehicles, and 8072 healthcare professionals, including a supporting staff comprising 5548 paramedics and EMTs, 761 pre-hospital trained medical assistants, and 59 physicians [3]. In 2016, the SRCA responded to 439,038 emergencies throughout the KSA [3].

However, the current EMS service model in the KSA is no longer the responsibility of SRCA; rather, it is the responsibility of the healthcare sector (public or private). For example, the General Directorate of Emergency and Disaster and Ambulance Services is responsible for all IFTs within MoH medical centers. Non-MoH institutes provide EMS to their catchment areas, but that represents a very small segment of the population. The private sector has no pre-hospital emergency care involvement and provides minimal services to patients who require non-critical transportation services, appointments for bed-ridden patients, hemodialysis, and discharges. Moreover, there are no specific regulations or scope of practice that oversee the provision of pre-hospital care, but efforts are ongoing to establish these regulatory documents and practices.

## Emergency medicine specialty training and education

### History

Prior to 1990, emergency medicine (EM) as a specialty was non-existent; instead, the departments of medicine, surgery, pediatrics, and obstetrics and gynecology treated their own emergencies in the emergency departments (EDs). In the 1990s, two major hospitals in Riyadh, King Faisal Specialist Hospital and the Ministry of National Guard Health Affairs Hospital, introduced American models of EDs. However, American contractors and expatriates operated these hospitals, and the entire emergency medical staff was composed of non-nationals. To increase the number of nationals in their EDs, these two hospitals established scholarships to send physicians to study EM as a specialty in Canadian and American universities. The returning graduates observed the need to start a national EM program, so they applied to the SCFHS to accept EM as a specialty based on a North American curriculum for residency training. At the time, the SCFHS determined the field was not ready to start its own EM Board, so they applied to well-established medical boards to accept EM as part of their specialty. The Surgical Scientific Board welcomed them with the help of the head of Emergency Department at King Khalid University Hospital. In 2000, EM was approved as a specialty under surgery, a coordination committee for EM was formed, and residency training of its first batch of four residents began. The training was provided in both the Health Affairs and in King Faisal Hospitals in Riyadh. In 2005, the SCFHS approved EM as an independent specialty and the Saudi Board of Emergency Medicine (SBEM) was formed. To this day, the SBEM is mandated to oversee and accredit EM residency programs, which now 22 different training centers across the kingdom.

### Residency training: general structure and curriculum

Medical specialty training in Saudi Arabia evolved in non-academic healthcare institutes providers, leading to significant discrepancies in training and certification until the SCFHS was established. The SCFHS has several bodies responsible for training and certification such as the SBEM, which approves the curriculum and training standards. Additionally, an EM exam committee independent of the SBEM administers board certification exams and reports to a central exam committee. Moreover, all the site program directors serve on regional supervisory committees to synchronize the training in all the sites and ensure they meet the national standards.

SBEM residency training is a structured 4-year postgraduate training program in emergency medicine, supervised and regulated by the SCFHS. The residency-training program provides the trainees to acquire the following competencies and function effectively as per the CanMEDS roles framework competencies. The 4-year residency-training program is divided into two parts: junior residency (the first 2 years) and senior residency (the final 2 years). During the senior residency years, residents are allocated to various subspecialties in EM in addition to seven rotations per year training in the ED. Residents are required to complete the allocated rotations satisfactorily for a given year and pass the end-of-year evaluation exams before moving from one academic year to the next.

After successful completion of all program requirements and obtaining the Final In-Training Evaluation Report, candidates receive a training completion certificate issued by the regional supervising training committee. The candidate is then eligible to take the Saudi Board Certification Final Examination in EM. Successful candidates receive the SBEM certificate and are licensed as a senior registrar by the SCFHS and begin independently working as EM attending physicians or consultants [10, 11].

### Hajj Mission Rotation: a unique aspect of EM residency training in the KSA

Although the SBEM curriculum is based on the North American model, some aspects of training are particular to the KSA, such as the Hajj rotation during which residents provide medical care to the 1–2 million Muslim pilgrims of different ages, nationalities, and ethnic backgrounds. The workforce is recruited from all over the KSA, including EM residents who spend a mandatory 1-month rotation in Hajj during their senior year, called the Hajj Mission Rotation. Hajj presents a unique opportunity for EM residents to gain experience in multiple aspects of healthcare service while meeting the medical needs of the pilgrims. They learn how to deliver high-quality medical care with limited resources and considerable language barriers [12, 13].

### Additional educational and professional development opportunities in EM

#### Fellowships in EM

The first fellowship to be established was the pediatric EM fellowship, which is a 2-year program that accepts graduates of both EM and pediatric boards. It began in 2006 and has several approved training sites across the KSA, all of which have fully dedicated pediatric emergency departments. The goal of this fellowship is to prepare graduates for all the possible emergencies seen in children aged 0–14 years, which is the upper limit for pediatric patients in the KSA, building on the pediatric training the EM residents received during residency. Two new fellowships specially designed for EM graduates are provided by the ED of Ministry of National Guard Health Affairs Hospital: a 2-year combined EMS and Disaster Management fellowship and a 1-year Emergency Ultrasound fellowship. The first prepares graduates to become competent directors of EMS departments as well as disaster and mass casualty response teams. The second prepares graduates with the competencies required to become bedside ultrasound directors, trainers, and quality assurance specialists for EDs.

#### Diploma in EM

The need for trained EM providers is huge but the number of SBEM graduates is still not enough to cover many regions in Saudi Arabia, especially rural and peripheral areas. A 2-year diploma in EM was started in 2017. It is a postgraduate training program with 13 blocks per academic year in which the trainee undergoes in clinical rotations similar to the first 2 years of the SBEM in addition to the Hajj rotation. After successful completion of 2 years of training and passing the final oral and written exams, the trainee will be recognized as a registrar by the SCFHS [11].

#### Boot camp courses for GPs working in peripheral non-academic EDs

The MoH, through its Emergency Departments Support Program (EDSP), has implemented an organized education outreach program, also called a boot camp course, for the education and training of general practitioners (GPs) working in peripheral EDs with the aim to improve and update the current practice of GPs regarding the most recent EM research.

#### Practice of emergency medicine

The SCFHS identifies EM (consultant) attending as a board-certified emergency physician who completed residency training in EM. Practicing EM attending physicians received their residency training locally, in the SBEM, or internationally in different countries such as the USA, Canada, Australia, South Africa, and France. Another subset of EM attending physicians is certified by the Arab Board of Emergency Medicine, which is regulated by the Arab Board of Health Specialization. Still, in the KSA, there is a massive need for certified EM physicians. To date, some hospitals and even cities still do not have a certified emergency physician working in the EDs and GPs or physicians who are internal medicine or general surgery training are acting as ED team leader.

#### Simulation in emergency medicine training

In the KSA, simulation-based learning has begun to be utilized by some academic hospitals with institutional capabilities and resources for simulation education. Bootcamps for emergency medicine residents are organized yearly to deliver a focused training for major EM procedures using simulation. A SCFHS project to integrate simulation-based learning in residency training, including EM residencies, is currently underway. A focus group conducted educational needs assessment to develop specialized simulation-based courses. Courses are designed and developed by EM physicians with medical education and simulation background. The program started in cities that have resources for running simulation courses, and have the opportunity to expand further. For instance, a mobile simulation truck is developed for future use to conduct simulation courses in the peripheral facilities without simulation resources, which holds promise for the improvement of EM training in underserved regions.

#### Societies, organizations, and conferences

The only society for EM physicians in the KSA is SASEM, which is affiliated with the SCHS. It was formed in 2008 and is run by a board of emergency physicians. It provides working groups for EMS, ultrasound, disaster management, toxicology, EM nursing, trauma, and critical care. It also runs the annual SASEM conference hosted in a variety of cities across the country, which is the main gathering of EM physicians in the KSA. Other notable symposiums include the Saudi Emergency Medicine Assembly (SEMA) and Best of EM, which are organized annually by groups interested in education. The training sites offer a large number of workshops specific to the practice of EM in the fields of bedside ultrasound, airway management, EM and surgical procedures, resuscitation, and emergency radiology. There is Saudi Association of EMS affiliated with SCFHS.

### Emergency medicine practice environment

The work environment in ED across the KSA is suboptimal in the majority of all non-academic and rural EDs. Among the reasons for this suboptimal quality is that the EDs are functioning in an outdated way in which patients are mainly treated by non-emergency trained physicians from other medical fields, such surgery, pediatrics, and general practice, who acquire their emergency skills through unsupervised experiences. Another reason is the fact that the ED is considered a temporary station for new medical staff before being assigned to their designated floors and, in some occasions, is a place where underperforming medical staff is assigned. Last but not least is the lack of awareness among the healthcare system’s leadership on the importance of a specialized ED, leading to under-budgeting and under-resourcing.

Academic EDs and hospitals with North American ED models are becoming increasingly attractive, competitive work environments for new graduate medical students, emergency residents, and qualified emergency physicians because of their high-quality care, efficiency, safe triaging, organized patient flow, cutting-edge technology, and the presence of qualified senior staff. However, other than these prestigious institutions, most emergency healthcare in Saudi Arabia is provided by physicians with little to no post-graduate training or who have been trained in other medical specialties [14, 15]. Moreover, as in other countries in the world, deficiency in nursing in Saudi is a serious challenge, with critical care and emergency nursing posing the hardest recruitment challenges [15]. Additionally, the number of nurses who are Saudi nationals is low, creating a dependence on expatriate nursing. The shortage of nurses and physicians in the ED has been shown to be a significant issue leading to overcrowding [15].

Relatively speaking, EM in the KSA is in better condition in relation to other countries in the Middle East and ranks highly in tertiary care centers [16]. It was also the 1st country in the region to establish a specialty certification in the form of the Saudi Board of Emergency Medicine, developed in 2001 by North-American-trained Saudi Physicians based on North American standards, and in 2007 the Saudi Society of Emergency Medicine was established to propagate and legislate the specialty [7]. The Saudi Board has also excelled in supporting the development of the Arab boards of emergency medicine across the Middle East [7].

## Challenges and proposed solutions

Strengthening of emergency care is an important step on the path to universal health coverage and is endorsed by WHO as “an essential part of integrated healthcare delivery” [17]. Moreover, both the Saudi National Transformation Program 2020 plan and the Saudi Vision 2030 plan have identified problems with the current healthcare system, including the burden faced by EDs, and have proposed targets for improving access to and quality of healthcare in the KSA, particularly primary and preventative care.

### Current challenges

The KSA is not an exception from the rest of the world facing serious public health concerns and has seen an increased chronic diseases burden. Current evidence has indicated that KSA has the 7th highest rate of diabetes mellitus (DM) in the world [18, 19] and has also experienced markedly increased rates of hypertension and coronary heart disease [9, 20]. In addition to increased chronic disease burden, the current system has seen a dramatic rise in the number of ED visits, leading to a dramatic increase in lengths of stay (LOS) for ED patients, which ultimately leads to ED overcrowding. Overcrowding has the potential to compromise patient care and is considered one of the most challenging problems facing EDs every day. In fact, 50% of medical directors surveyed in Riyadh, KSA, reported that ED crowding is a major problem [21]. Poor hospital flow is unacceptable in modern healthcare systems, so data-driven, evidence-based policies are needed.

Currently, the ED functions as the main gateway to medical care in the KSA. While the healthcare system is transitioning from acute care to preventative and primary care, that requires a great deal of health policy research and effortful implementation that can anticipate and proactively address issues based on the trends of other external institutions and from evidence from within the Saudi hospital system. Additionally, EDs in some regions are more strained than others. For instance, a recent study revealed that emergency services in the Abha district of southwestern KSA faced a lack of some essential equipment and drugs [14]. The Abha district study also revealed that the greatest continuing medical education need for physicians in this district was the management of cardiovascular emergencies [14].

A study by Aalam et al. found that although EM residency training programs in the KSA are all of reasonably high quality as measured by the Postgraduate Hospital Educational Environment Measure (PHEEM) instrument, compared to American EM residents, Saudi EM trainees reported feeling less competent in managing multiple traumas and perceived faculty supervision and faculty interest in education as less strong; they also reported seeing more patients per hour than US residents [10]. These findings may result from differences in training techniques between the two countries, including less formal didactics and simulation experience in KSA and more duty hour regulations in the USA. A few changes, including the inclusion of simulation in the EM curriculum in Saudi Arabia as well as changes in policies and regulations, might increase the Saudi residents’ opinion of their residency training experience [10].

### Proposed solutions

To address the problems in Saudi EDs, a number of solutions have been proposed and implemented with varying degrees of success. The common thread that connects all the proposed solutions shared the goal of reserving the ED for truly emergent health issues rather than the current trend of patients using the ED as the main gateway to hospital admission.

#### Access to healthcare

One way to achieve this goal involves other providers filling the existing gap in care, particularly primary care physicians (PCPs), urgent care facilities, home healthcare, and rehabilitation services. Urgent care can provide quick access to medical care, including after-hours (when a patient needs healthcare but cannot see their PCP and is faced between going to the ED or waiting until morning to use primary healthcare services). Urgent cares can also release some of the burden placed on EDs and fill in during PCPs’ off hours, which can be quite useful to patients. Moreover, improved primary care and referral systems (i.e., follow-up) can relieve some of the pressure placed on EDs. Understanding the barriers and enablers to accessing primary healthcare will likely help reduce inequalities in access to and utilization of primary healthcare, especially suburban and rural populations. Strengthening and monitoring of the follow-up system and the communication between PCPs and EDs is one way to overcome the barriers faced by PCPs. For example, among patients who reported being frequent users of EDs, approximately one third cited the “availability of modern diagnostic equipment” (37.3%) and “to avoid taking time off from work” (33.0%) as reasons for using EDs [22]. Findings like this suggest that better resource in the hands of PCPs and more available hours (whether met by PCPs or urgent care facilities) are two of the key enablers that could encourage more patients to seek primary care rather than always immediately going to the ED. Finally, home healthcare and rehabilitation services are also underdeveloped in the KSA and, as such, present an opportunity to expand services and relieve the burden faced by hospitals and particular EDs.

#### Quality of healthcare

In addition to increasing access to healthcare, the KSA has many opportunities to improve the quality of healthcare provided. One way to improve healthcare quality is to enhance patient flow coordination and facilitation. This enhancement could be achieved through measures such as establishing the role of a “bed czar” or patient flow manager. This person would be responsible for ensuring timely transfer of ED patients to assigned inpatient beds. Assigning a dedicated nurse with admissions/discharge/transfer duties who is specifically responsible for facilitating discharges to accelerate available beds for admissions. Furthermore, the development of accelerated triage and registration processes based on patient’s acuity could reduce waiting times [23]. Along the same lines, early discharge can improve flow and can be achieved in a number of ways, including:

- Initiation of preliminary discharge by designating patients for early discharge the next day.
- Establishing a discharge room/lounge for inpatients who have been discharged and are awaiting transportation, medications or education.
- Establishing a discharge coordinator position to coordinate procuring information that is required to discharge the patient.
- Implementing monetary incentives and nonmonetary incentives for physicians and nurses to promote efficient and early discharge of patients who are ready to go home [23].

Flow is of the utmost importance to high quality are and is prioritized in most quality assurance (QA) objectives. Flow, or lack thereof, is also a factor in waiting time and triage procedures in EDs Waiting times can have a dire impact on high acuity patients. Long waiting time is associated with delays in time-sensitive treatments for serious conditions typically requiring admission [24]. Many EDs use a physician-in-triage (PIT) process to overcome the negative impacts of patient waiting time on patient care quality and efficiency.

Diversion management and reduction is still another way the quality of healthcare can be improved. This improvement requires establishing new protocols and monitoring systems to allow early warning when the system is approaching its maximum capacity and its threshold for diversion. Additionally, this would necessitate developing a hospital-wide diversion response protocol to focus existing resources on facilitating all appropriate patient discharges in a timelier manner. Finally, the creation of a community-wide diversion plan in collaboration with local hospitals and the community’s emergency medical services unit could help establish a common protocol for hospitals going on and off diversion or bypass [23].

#### Quality of and access to EMS services

In addition to enhancing access to healthcare through diversification of options and emphasis on primary care, improved provision of healthcare in the KSA can also be achieved through better EMS education, training and staffing. One problem with EMS is that the majority of EMS calls are for relatively minor complaints or non-medical complaints. For example, a recent study of EMS transport calls found that an alarming 25% of calls made during the study period were reported as non-transported calls. Of those, 70% stemmed from refusal by patient while 22.4% of were canceled by dispatch [25].

Gender also plays an important role in the quality of EMS care. In a conservative nation like the KSA, the care provided to men and women can be disparate. For example, the ratio of males to females in EMS calls was 2:1; males accounted for 722 calls (51.9%) while females accounted for only 325 (23.4%). In a significant number of cases, the gender was not listed [25]. The lopsided ratio is likely because a vast majority of females arrived by private vehicles. In light of this disparate ratio and with the recent change in the KSA allowing women to drive, the need for female EMS personnel has become abundantly clear.

## Conclusion

The KSA has reached nearly 100 years of providing healthcare to its people. However, the healthcare system in the KSA only reached modern standards of quality in the early 2000s. For the past 10+ years, the healthcare system has rapidly improved, reaching the level of quality achieved by many healthcare systems in the West. In the coming years, an increasing emphasis on primary care is expected to begin to ameliorate the common problem of EDs being treated as the gateway to the hospital. Moreover, promising trends in the development of simulation-based education, improvements in residency programs and curricula, and diversification of healthcare providers indicate that the rapid progress experience over the course of the last decade is poised to continue for the next decade and beyond. Roadmaps like the National Transformation Plan 2020 and the Saudi Vision 2030 have also begun to pave the way for future developments. It is now the task of Saudi EM physicians and healthcare policymakers to conduct practical research and implement data-drive, evidence-based policies, and procedures to continue to guide efforts to move towards a more preventive and primary care healthcare model.

## Data Availability

Not applicable.

## Abbreviations

CME: Continued Medical Education
ED: Emergency Department
EM: Emergency medicine
EMS: Emergency medical services
EMT: Emergency medical technician
KSA: Kingdom of Saudi Arabia
MNG: Ministry of National Guard
MoD: Ministry of Defense
MoE: Ministry of Education
MoH: Ministry of Health
MoI: Ministry of Interior
PHEEM: Postgraduate Hospital Educational Environment Measure
SASEM: Saudi Society of Emergency Medicine
SBEM: Saudi Board of Emergency Medicine
SCFHS: Saudi Commission for Health Specialties
SRCA: Saudi Red Crescent Association/Authority
